# Supplementary material for: Concurrent validity of novel smartphone-based apps monitoring barbell velocity in powerlifting exercises
Source: PLoS One. 2024 Nov 19;19(11):e0313919. doi: 10.1371/journal.pone.0313919 (PMC11575817; doi:10.1371/journal.pone.0313919)
Supplement: Attachment — Submitted filename: Response to Reviewers.pdf [file pone.0313919.s001.pdf]

Dear Dr. Greco and reviewers,

We would like to thank you for the constructive feedback on our manuscript. Your comments and suggestions have been invaluable in helping us refine our work. We have carefully considered each point raised and are pleased to provide our responses to them below.

Colour Code:
Feedback
Response

Reviewer #1: The article was properly prepared in accordance with scientific research standards. The authors used appropriate research methodology. The reservations that may arise concern only a small number of sources used in the bibliography (it would be worth supplementing it in subsequent articles with publications by Prof. Michał Wilk or Prof. Michał Krzysztofik). The topic is extremely interesting due to the usefulness of the results achieved in the process of sports training or training of people practicing strength sports recreationally.
One article from Prof. Michal Krzysztofik has been added to the bibliography: “The bench press exercise performed with increased range of motion allows for greater bar velocities.” This article adds substantial value to the discussed kinematic properties of the bench press in lines 121-122.

Furthermore, two article from Basil Achermann and colleagues have been added to the bibliography: “Velocity-Based Strength Training: The Validity and Personal Monitoring of Barbell Velocity with the Apple Watch” & “Velocity-based strength training: comparing duration and mean velocity to predict the maximum number of repetitions in a set”. These articles add substantial value by adding that smartwatches also can serve similar functions as the SAs in lines 58-59 and discussing VBT variables beyond mean and peak velocity in lines 40-42.

Reviewer #2: Review of “Concurrent validity of novel smartphone-based apps monitoring barbell velocity in powerlifting exercises”

This paper presents a valuable effort to examine the validity of three smartphone apps designed to quantify the velocity of a barbell during the three major powerlifting exercises. The study’s approach, which includes the use of a linear position transducer and a comparison with the Vicon system as the gold standard, is commendable. Additionally, it’s great to see that the study included both male and female powerlifters, providing a more comprehensive analysis.

Abstract:

To strengthen the abstract, it would be beneficial to include data on the typical mean velocity or the relative size of the RMSE compared to the mean velocity. This additional information would help readers quickly grasp the significance of the findings.

The mean mean velocity has been added in lines 26-27 to the abstract in order to contextualize the findings.

Introduction:

While the introduction provides a solid foundation, the use of abbreviations could be made clearer. For instance, “MV” stands for mean velocity, but “MT” refers to an app, which may be confusing for readers.
For better readability the abbreviations MV and PV were removed.

•Line 42: The discussion around the different velocity parameters (mean velocity, peak velocity, etc.) could be elaborated to better reflect the ongoing debate in the field.
In order to adequately reflect the ongoing debate in the field a citation from Basil Achermann and colleagues has been added (“Velocity-based strength training: comparing duration and mean velocity to predict the maximum number of repetitions in a set”) and it was mentioned in lines 40-42 that more advanced parameters exist and have shown potential.

•Line 54: It might be worth mentioning that there are also smartwatch applications available that serve similar functions.
The information has been added in lines 58-59 together with a citation of Basil Achermann and colleagues: “Velocity-Based Strength Training: The Validity and Personal Monitoring of Barbell Velocity with the Apple Watch”.

Table 1:

The values in Table 1 would benefit from rounding to more meaningful figures. For example, 541.88±155.91 could be rounded to 541±156 to enhance readability and comprehension.

The values were rounded to more meaningful figures. The reasoning for each rounding are given below.

Age & Height -1 decimal: Both age and height were recorded without decimals. It was decided to round the mean and SD to one decimal to give a more meaningful overview over the study participants.

Body mass - 1 decimal: Body mass was recorded with 1 decimal and is rounded to such.

Absolute Powerlifting Performance - 1 decimal: The squat, bench press, deadlift are typically recorded in 2.5kg increments as these are the increments a lifter can choose from in competition. For local, national or international records a lifter can choose their weight in 0.5kg increments. Therefore the weight was recorded with 1 decimal and is rounded to such.

Relative Powerlifting Performance - 2 decimals: While some publications round the relative strength performance to one decimal (13) the vast majority of similar publications round the

relative strength performance to two decimals. This can be seen in the publications 3,4,6,8,11,20 in our bibliography. Therefore we chose to round these values to 2 decimals.

Wilks & IPF GL - 2 decimals: Both Wilks and IPF GL are typically recorded to two decimals in competition. They were recorded to two decimals in this study and are rounded as such.

Methodology:

•Line 169: A more detailed description of the barbell fixation would be helpful. Including a picture of the setup would significantly improve clarity.
Further details were added in lines 175-178.

•Additionally, please provide information on the frequency of the sensors used in the iPhone.
Wherever known, the frequency of the SAs is already provided. For QW the videos were recorded with the native camera application and a frequency of 60FPS was chosen (the highest possible frequency as recommended by the developer). For MT and ML the videos were recorded with the SAs themself. It is not known which frequency/FPS the developer of these two SAs use within their application. With ML it is possible to export the raw position data but it cannot be said with certainty that the data output is identical with the FPS used by the SA.

•Line 207: Clarifying the thresholds and any other steps involved in data processing would enhance the transparency of the methodology.
All steps and thresholds can already be found in the manuscript. The instantaneous velocity threshold was set at 0.02m/s and the ROM threshold was set to 10cm. No further thresholds were used. For full transparency the Python script which was used can be found in the online repository. Further explanation was added in lines 218-220.

Figures:

The resolution of the figures, particularly Figure 6, needs improvement as it is currently difficult to read. High-quality visuals are crucial for effective communication of the data.
The figures submitted to the journal were of high resolution (resolution: 600 dpi, dimensions: 2125x2125, 2125x4251, 2834x4251). The low resolution might be caused by the journal providing a compressed version of the figures. We will check with the academic editor to make sure all figures have adequate resolution.

Results Section:

The beginning of the results section currently reads more like a continuation of the methods. Revising this to make it more reader-friendly would improve the flow of the paper.
While we appreciate the feedback, we decided to leave the beginning of the results section as is. We explained the protocol and how the data was processed in the methods section. The first part of the results section yields descriptive results about how many repetitions were recorded and for what reasons the actual amount of repetitions recorded differs from the hypothetical

amount of repetitions recorded. Therefore, we consider the beginning of the results section an integral component of the results section. We hope for your understanding.

•Line 248: Further clarification is needed here. Could you explain the reasoning behind this choice?
Further details were added in lines 256 -259.

•Text and Figure 2: It would be helpful to present the data only once to avoid redundancy. Including ghost repetitions in Figure 2 would also provide a clearer representation of the findings.
We understand the concern regarding redundancy, but we would like to clarify the distinction between the text and Figure 2. In the text, we present absolute frequencies, while the figure shows relative (percentage) frequencies. This allows us to provide complementary perspectives on the data, each emphasizing different aspects.

Regarding the inclusion of ghost repetitions in the figure, they occur with such a low frequency in the dataset that visualizing them would either render the representation unnecessary (since there are only two non-zero values, both very small) or require manipulating the axis scale to focus on the 0-10% range. The latter approach may lead to potential misinterpretation of the findings.

We believe that presenting the data in this manner provides a clearer understanding of the distribution without distorting the visual impact of rare events. Therefore, we would recommend keeping the figure as it stands.

Currently, the results section blends text and figure legends. If the legends are removed, there is little information left in the text. Including more detailed information directly in the results section would enhance its substance.
We would like to explain that the current structure of the results section, which blends text and figure legends, was deliberately chosen to ensure clarity and readability. Given that we are presenting results from 3 exercises, 4 tools, and 5 statistics, a more detailed listing of all results in the text would, in our view, make the article harder to follow and overly dense.

Additionally, by structuring the results this way, we aim to avoid redundancy, which aligns with the reviewer's later comment regarding minimizing unnecessary repetition. Furthermore, figures in online publications are typically displayed directly in the text where they are referenced, as is common in journal formats such as those found in PLOS One (https://journals.plos.org/plosone/article?id=10.1371/journal.pone.0268074).

We believe this approach maintains a balance between providing sufficient detail and ensuring that the results are presented in an accessible manner.

Discussion:

Lastly, the addition of two models to interpret the data seems to introduce some confusion. It would be beneficial to ensure that these models align closely with the study's objectives, or to reconsider their inclusion if they do not add substantial value.

We would like to state that it is quite common in validation studies to employ multiple models for data analysis. Many studies combine techniques such as paired samples t-tests or repeated measures ANOVA with regression analysis or Bland-Altman analysis. This can be seen in articles 3, 4, 6, 8, 11, 17, 20, 21, 24, and 25 in our bibliography, which all employ multiple models to ensure a robust and comprehensive analysis.

The models we use are essentially equivalents to models used in regression analysis (model 1) and paired samples t-test (model 2), but both are corrected for a hierarchical data structure. This correction is necessary to avoid violating the assumption of independence.

Furthermore, the inclusion of model 2 was crucial for determining the SMB using Bayesian methods, which allows us to present uncertainty intervals for the point estimates. Given that our study is exploratory in nature, maintaining a Bayesian approach throughout was important to us.

We believe that the use of these two models aligns with the study's objectives and adds value by ensuring a thorough and appropriately structured analysis of the data.

I hope these suggestions help in refining your manuscript. With these revisions, the paper has the potential to make a significant contribution to the field.
